# Sexual and gender minority youth in Canada: An investigation of disparities in positive mental health

**DOI:** 10.17269/s41997-024-00931-4

**Published:** 2024-09-25

**Authors:** Sonia Hajo, Colin A. Capaldi, Li Liu

**Affiliations:** 1 Centre for Surveillance and Applied Research, Public Health Agency of Canada, Ottawa, ON Canada; 2 Department of Epidemiology, Biostatistics and Occupational Health, McGill University, Montreal, QC Canada

**Keywords:** Mental health, Adolescent well-being, Happiness, Satisfaction, Sexual and gender minorities, Health inequalities, Santé mentale, bien-être des adolescents, bonheur, satisfaction, minorités sexuelles et de genre, inégalités en matière de santé

## Abstract

**Objectives:**

While studies indicate that 2SLGBTQ + youth are more likely to experience negative psychological outcomes compared to their heterosexual and cisgender peers, less is known about the positive mental health (PMH) of 2SLGBTQ + youth in Canada. To fill this gap, we investigated disparities in PMH by self-reported sexual attraction among 15‒17-year-olds and gender modality among 12‒17-year-olds.

**Methods:**

We analyzed data from youth respondents in the 2019 Canadian Health Survey on Children and Youth. We obtained estimates of average life satisfaction and high self-rated mental health, happiness, autonomy, competence, and relatedness for youth with an exclusively heterosexual attraction and youth with a minority sexual attraction (those exclusively attracted to the same gender, and those attracted to both females and males), and for cisgender and gender minority youth. Regression analyses were conducted to test for disparities on each PMH outcome.

**Results:**

Compared with exclusively heterosexual youth, sexual minority youth reported lower life satisfaction and were less likely to report high self-rated mental health, happiness, autonomy, competence, and relatedness. Significant disparities were more consistently found for youth attracted to both females and males than youth exclusively attracted to the same gender. Gender minority (versus cisgender) youth also reported lower average life satisfaction and were less likely to report high self-rated mental health, happiness, autonomy, competence, and relatedness.

**Conclusion:**

Although this study provides evidence for the presence of disparities in PMH, its strength-based focus on PMH also documents the presence of well-being among many sexual and gender minority youth in Canada.

## Résumé

**Objectifs:**

Alors que les études indiquent que les jeunes 2ELGBTQ + sont plus susceptibles de subir des conséquences psychologiques négatives que leurs pairs hétérosexuels et cisgenres, nous en savons moins sur la santé mentale positive (SMP) des jeunes 2ELGBTQ + au Canada. Pour combler cette lacune, nous avons étudié les disparités en matière de SMP en fonction de l’attirance sexuelle déclarée par les jeunes concernés de 15 à 17 ans et de la modalité de genre déclarée par les jeunes de 12 à 17 ans.

**Méthodes:**

Nous avons analysé les données relatives aux jeunes répondants dans le cadre de l’Enquête canadienne sur la santé des enfants et des jeunes de 2019. Nous avons obtenu des estimations de la satisfaction moyenne à l’égard de la vie et de la santé mentale autoévaluée élevée, du bonheur, de l’autonomie, de la compétence et de l’appartenance sociale pour les jeunes ayant une attirance exclusivement hétérosexuelle et les jeunes ayant une attirance sexuelle minoritaire (ceux qui sont attirés exclusivement par le même genre et ceux qui sont attirés à la fois par les femmes et les hommes), ainsi que pour les jeunes cisgenres et les jeunes appartenant à une minorité de genre. Des analyses de régression ont été effectuées pour vérifier l’existence de disparités pour chaque résultat en matière de SMP.

**Résultats:**

Par rapport aux jeunes exclusivement hétérosexuels, les jeunes appartenant à une minorité sexuelle ont déclaré une plus faible satisfaction à l’égard de la vie et étaient moins susceptibles d’évaluer leur santé mentale, leur bonheur, leur autonomie, leurs compétences et leur appartenance sociale comme étant élevés. Des disparités significatives ont été plus régulièrement constatées chez les jeunes attirés à la fois par les femmes et les hommes que chez ceux exclusivement attirés par le même genre. Les jeunes appartenant à une minorité de genre (par rapport aux jeunes cisgenres) ont également fait état d’une plus faible satisfaction moyenne à l’égard de la vie et étaient moins susceptibles d’évaluer leur santé mentale, leur bonheur, leur autonomie, leurs compétences et leur appartenance sociale comme étant élevés.

**Conclusion:**

Bien que cette étude fournisse des preuves de l’existence de disparités en matière de SMP, sa focalisation sur les points forts de la SMP documente également la présence du bien-être chez de nombreux jeunes des minorités sexuelles et de genre au Canada.

## Introduction

Sexual and gender minority (SGM) individuals make up a relatively sizeable portion of youth in Canada, with approximately one in five youth aged 15–17 years reporting a sexual attraction that is not exclusively heterosexual and a half percent of youth aged 12–17 reporting a gender identity that differs from their sex assigned at birth (Wang et al., 2023). Research indicates that these individuals are at a higher risk of negative psychological outcomes relative to heterosexual and cisgender youth (Plöderl & Tremblay, 2015; Wittlin et al., 2023). For instance, meta-analyses reveal that sexual minority youth have a higher risk of depression (Lucassen et al., 2017), suicidality (di Giacomo et al., 2018), non-suicidal self-injury (Liu et al., 2019), and eating disorder–related behaviours (Cao et al., 2023) compared to heterosexual youth. Meta-analytic estimates also suggest a high prevalence of suicidality and non-suicidal self-injury among gender minority youth (Liu et al., 2019; Surace et al., 2021). Disparities in these types of negative psychological outcomes have been observed among SGM youth in Canada as well (e.g., Blais et al., 2015; Kingsbury et al., 2022; Veale et al., 2017). In line with minority stress theory (Frost & Meyer, 2023; Meyer, 2003), these disparities may stem from the additional distal stressors (e.g., violent victimization) and proximal stressors (e.g., expectations of rejection) experienced by SGM youth (de Lange et al., 2022; Dürrbaum & Sattler, 2020).

The examination of negative psychological outcomes in the aforementioned research only captures part of how SGM youth are feeling and functioning. Investigating positive mental health (PMH) outcomes shifts attention to a strength-based perspective and provides opportunities to document well-being among SGM youth. PMH is multifaceted; it encompasses various aspects of mental well-being, including how good a person is feeling emotionally and about their life (i.e., hedonic well-being), and how well a person is functioning psychologically and socially (i.e., eudaimonic well-being; Keyes, 2013). The former includes elements like happiness and life satisfaction, while the latter can include (among other elements) having a sense of competence or mastery over one’s environment, having a sense of autonomy or self-determination, and having a sense of relatedness or connectedness to others (Huta & Waterman, 2014; Ryff, 1989). An expanded focus on the PMH of SGM youth has been identified as an important area for future research (Fish, 2020). This is especially relevant in Canada given the limited number of studies that have reported on PMH disparities by SGM status among youth in particular.

Findings from British Columbia, Canada, in 2008 and 2013 show that sexual minority (versus heterosexual) youth were less likely to report that they usually felt good about themselves and that they could do things as well as other people (Watson et al., 2018). Moreover, in the 2019 Canadian Trans and Non-binary Health Youth Survey, only 16% of gender minority youth and young adults reported “excellent” or “good” mental health and 22% reported never being satisfied with their life (Taylor et al., 2020). These results should be interpreted with caution, however, as the questions are not identical to those used in national population-based surveys and/or convenience sampling was used to recruit participants.

The nationally representative Canadian Health Survey on Children and Youth (CHSCY) conducted by Statistics Canada (2019) provides an opportunity to more comprehensively examine this topic as it included questions about sexual attraction, sex, gender, and numerous outcomes that are used by the Public Health Agency of Canada to monitor youth PMH in its Positive Mental Health Surveillance Indicator Framework (Centre for Surveillance and Applied Research, n.d.). The objectives of this study were to (1) estimate PMH among SGM youth in Canada and (2) investigate potential disparities in PMH that SGM youth may experience relative to heterosexual and cisgender youth.

## Methods

### Data and participants

We analyzed cross-sectional data from youth from the 2019 CHSCY, which was collected by Statistics Canada from February 11 to August 2, 2019 (Statistics Canada, 2019). The target population of the 2019 CHSCY was individuals aged 1‒17 years who were living in any of the 10 provinces or 3 territories in Canada. Children and youth who were institutionalized, who were living in foster homes, or who were living on First Nations reserves and other Indigenous settlements were excluded from the 2019 CHSCY. The Canada child benefit was used for the sampling frame, which covers 98% of the population aged 1‒17 years in the provinces and 96% in the territories. The 2019 CHSCY was voluntary and completed online or over the telephone with an interviewer. The response rate for youth aged 12‒17 years was 41.3%, with a total of 11,077 out of 13,602 youth respondents (81.4%) agreeing to share their data with the Public Health Agency of Canada and Health Canada. Our study focused on these youth aged 12‒17 years for gender minority versus cisgender comparisons, but was restricted to youth aged 15‒17 years for comparisons by sexual attraction as that question was not asked to younger respondents.

### Measures

#### Positive mental health outcomes

All six PMH outcomes included in the youth version of the Positive Mental Health Surveillance Indicator Framework were assessed for respondents aged 12‒17 years in the 2019 CHSCY (Centre for Surveillance and Applied Research, n.d.). These outcomes are positively associated (Capaldi & Ooi, 2023; Lombardo et al., 2018), but attempt to capture some of the different aspects of hedonic and eudaimonic well-being (Huta & Waterman, 2014; Keyes, 2013; Ryff, 1989).

Self-rated mental health was measured by asking “In general, how is your mental health?”, with “Excellent”, “Very good”, “Good”, “Fair”, and “Poor” as response options. We dichotomously coded youth who responded “Excellent” or “Very good” as having high self-rated mental health; this can be considered an indicator of overall PMH. Similar single-item measures of self-rated mental health have been used for decades and have been associated with standardized multi-item mental health scales (Ahmad et al., 2014). Moreover, the Organisation for Economic Cooperation and Development (2023) recently recommended this as one measure that could be used to monitor population mental health consistently across countries.

Life satisfaction was measured by asking “Using a scale of 0 to 10, where 0 means ‘Very dissatisfied’ and 10 means ‘Very satisfied’, how do you feel about your life as a whole right now?” We examined average life satisfaction, which captures one aspect of hedonic well-being. There is a large amount of research on the validity of life satisfaction measures (Diener et al., 2013). Single-item measures of life satisfaction are already used to monitor well-being internationally (Organisation for Economic Cooperation & Development, 2020).

Another aspect of hedonic well-being that we examined was happiness, which was measured by asking “How would you usually describe yourself?”, with “Happy and interested in life”, “Somewhat happy”, “Somewhat unhappy”, “Unhappy with little interest in life”, and “So unhappy, that life is not worthwhile” as response options. We dichotomously coded youth who responded “Happy and interested in life” as having high happiness. This item is the emotion attribute from the Health Utility Index Mark 3 (Horsman et al., 2003), which has been shown to have convergent validity (Feeny et al., 2009).

Finally, aspects of eudaimonic well-being (i.e., psychological and social well-being) were assessed using the Children’s Intrinsic Needs Satisfaction Scale (Véronneau et al., 2005). This scale asks about sense of autonomy (e.g., “I feel free to express myself with my friends”), competence (e.g., “I feel I do things well at home”), and relatedness (e.g., “I like to be with my teachers”) with peers, at home, and at school. Response options include “1: Really false for me”, “2: Sort of false for me”, “3: Sort of true for me”, and “4: Really true for me”. We dichotomously coded individuals who had a mean score of 3 or higher on the six autonomy questions, the six competence questions, or the six relatedness questions as having high autonomy, high competence, or high relatedness, respectively. Previous research has validated this scale for use with youth in Canada (e.g., Capaldi & Ooi, 2023).

#### Gender modality

The gender modality of youth was determined based on the congruence or incongruence of their sex at birth and gender identity (Rioux et al., 2022). Specifically, youth aged 12–17 years were asked “What was your sex at birth?”, with “Male” and “Female” as response options. This was followed by the question “What is your gender?”, with “Male”, “Female”, and “Or please specify” as response options. When responses for sex at birth and gender identity were the same, individuals were coded as cisgender; when responses differed, they were coded as a gender minority.

#### Sexual attraction

Youth aged 15‒17 years indicated the degree to which they were sexually attracted to males and females. Response options included “Only attracted to males”, “Mostly attracted to males”, “Equally attracted to females and males”, “Mostly attracted to females”, “Only attracted females”, and “Not sure”. Individuals were coded as “only attracted to the opposite gender” if (1) their sex at birth and gender identity was male and they indicated that they were “Only attracted to females”, or (2) their sex at birth and gender identity was female and they indicated that they were “Only attracted to males”. Individuals were coded as “only attracted to the same gender” if (1) their sex at birth and gender identity was male and they indicated that they were “Only attracted to males”, or (2) their sex at birth and gender identity was female and they indicated that they were “Only attracted to females”. Finally, cisgender individuals were coded as “attracted to both males and females” if they were “Mostly attracted to males”, “Equally attracted to females and males”, or “Mostly attracted to females”. For broad comparisons, we also coded youth who reported being only attracted to the same gender or attracted to both males and females into an overall sexual minority group. We excluded youth who responded “Not sure” to the sexual attraction question and youth classified as a gender minority from the analyses involving sexual attraction.

#### Covariates

Sociodemographic characteristics that were included in this study as covariates were sex at birth (male, female), visible minority status (yes, no), household income quintile, and place of residence (population centre, rural area). The classification of youth as belonging to a visible minority group was based on the definition used in the *Employment Equity Act* (i.e., “persons, other than Aboriginal peoples, who are non-Caucasian in race or non-white in colour”; Statistics Canada, 2021a). Household income (before taxes and deductions) was either reported by the person-most-knowledgeable (typically a parent) or imputed by Statistics Canada if survey responses regarding income were missing. Based on postal codes, Statistics Canada classified individuals living in continuously built-up areas with populations of at least 1000 and population densities of at least 400 per km^2^ as living in population centres.

### Analysis

We conducted analyses in SAS Enterprise Guide version 7.1 (SAS Institute, Cary, NC, USA). All estimates were weighted by sampling weights provided by Statistics Canada; standard errors, coefficients of variation, and 95% confidence intervals (CI) were estimated using bootstrap resampling method with 1000 replications.

We obtained overall and sex-stratified estimates of mean life satisfaction and the prevalence of high self-rated mental health, happiness, autonomy, competence, and relatedness for youth who were only attracted to the opposite gender and youth with a minority sexual attraction. We also obtained overall estimates of these PMH outcomes specifically for youth who were only attracted to the same gender and youth who were attracted to both males and females. The six PMH outcomes were estimated for gender minority youth and cisgender youth as well.

We conducted regression analyses to examine whether the aforementioned PMH estimates significantly differed by sexual attraction and gender modality. Specifically, we used linear regression analysis for life satisfaction and logistic regression analysis for the four dichotomized PMH outcomes, with youth who were only attracted to the opposite gender as the reference group in the comparisons by sexual attraction and cisgender youth as the reference group in the comparisons with gender minority youth. We also conducted additional analyses that included covariates for the overall comparisons involving the broad sexual minority group to determine whether disparities in PMH were observed after statistically controlling for other sociodemographic characteristics. We identified statistically significant differences by unstandardized linear regression coefficients with 95% CIs that did not include 0 in the linear regression analyses and by odds ratios (OR) with 95% CIs that did not include 1.0 in the logistic regression analyses. We do not report sex-stratified estimates or adjusted regression analyses for gender minority youth or the specific sexual minority groups due to small sample sizes resulting in some unreleasable estimates and/or very wide CIs.

Following recommendations from the 2019 CHSCY User Guide, estimates with coefficients of variation greater than 15% and less than or equal to 25% are flagged with a “^C^”, and those greater than 25% and less than or equal to 35% are flagged with a “^D^”. These estimates should be interpreted with caution due to high sampling variability.

## Results

The distribution of sociodemographic characteristics, including sexual attraction and gender modality, is reported in Table 1.

**Table 1:** Sociodemographic characteristics, 2019 CHSCY

	Percentage (95% LCL, 95% UCL)
Sex at birth (*n* = 11,076)	
Male	51.3% (51.2, 51.4)
Female	48.7% (48.6, 48.8)
Visible minority (*n* = 11,040)	
Yes	29.3% (28.2, 30.4)
No	70.7% (69.6, 71.8)
Place of residence (*n* = 11,077)	
Population centre	81.8% (80.9, 82.7)
Rural area	18.2% (17.3, 19.1)
Sexual attraction (*n* = 5050)^a^	
Only attracted to opposite gender	82.6% (81.2, 84.0)
Sexual minority	17.4% (16.0, 18.8)
Only attracted to same gender	0.9% (0.6, 1.2)^C^
Attracted to both males and females	16.5% (15.1, 17.9)
Gender modality (*n* = 11,063)	
Cisgender	99.5% (99.3, 99.7)
Gender minority	0.5% (0.3, 0.7)^C^

Results are weighted. Sample sizes did not all equal 11,077 due to missing/not sure responses and the sexual attraction question not being asked of youth under 15 years old. *CHSCY*, Canadian Health Survey on Children and Youth; *LCL*, lower confidence limit; *UCL*, upper confidence limit

^a^Estimates differ from those reported in Wang et al. (2023) due to our exclusion from analyses of youth who responded “not sure” when asked about their sexual attraction

^C^Interpret estimate with caution because coefficient of variation is greater than 15 and less than or equal to 25

### PMH and sexual attraction

Comparisons of each PMH outcome by minority sexual attraction versus exclusively heterosexual attraction are reported in Table 2. Sexual minority youth reported significantly lower average life satisfaction (6.9 versus 7.9; *b* =  − 0.9; 95% CI, − 1.1 to − 0.7) and were significantly less likely to report high self-rated mental health (32.4% versus 63.6%; OR = 0.3; 95% CI, 0.2 to 0.3), happiness (43.4% versus 61.5%; OR = 0.5; 95% CI, 0.4 to 0.6), autonomy (70.5% versus 82.0%; OR = 0.5; 95% CI, 0.4 to 0.7), competence (72.8% versus 83.5%; OR = 0.5; 95% CI, 0.4 to 0.7), and relatedness (82.7% versus 88.5%; OR = 0.6; 95% CI, 0.5 to 0.8) than youth who were only attracted to the opposite gender. These disparities remained statistically significant when controlling for other sociodemographic characteristics and when stratified by sex, except for high relatedness among females with a minority sexual attraction versus an exclusively heterosexual attraction.

**Table 2:** Positive mental health outcomes in youth aged 15–17 years by minority sexual attraction versus exclusively heterosexual attraction, overall and stratified by sex, 2019 CHSCY

Positive mental health outcome	Overall			Female		Male	
	Prevalence (95% LCL, 95% UCL)	Unadjusted OR (95% LCL, 95% UCL)	Adjusted OR (95% LCL, 95% UCL)	Prevalence (95% LCL, 95% UCL)	Unadjusted OR (95% LCL, 95% UCL)	Prevalence (95% LCL, 95% UCL)	Unadjusted OR (95% LCL, 95% UCL)
High self-rated mental health							
Sexual minority	32.4% (28.1, 36.7)	**0.3** **(0.2, 0.3)**	**0.3** **(0.2, 0.4)**	26.1% (21.1, 31.0)	**0.3** **(0.2, 0.4)**	44.4% (36.6, 52.2)	**0.3** **(0.2, 0.5)**
Only attracted to opposite gender	63.6% (61.6, 65.6)	Reference	Reference	55.6% (52.6, 58.7)	Reference	70.2% (67.6, 72.7)	Reference
High happiness							
Sexual minority	43.4% (38.9, 47.9)	**0.5** **(0.4, 0.6)**	**0.5** **(0.4, 0.6)**	41.0% (35.5, 46.5)	**0.5** **(0.4, 0.7)**	48.0% (40.2, 55.8)	**0.5** **(0.3, 0.7)**
Only attracted to opposite gender	61.5% (59.5, 63.6)	Reference	Reference	56.2% (53.1, 59.4)	Reference	65.9% (63.1, 68.6)	Reference
High autonomy							
Sexual minority	70.5% (66.4, 74.6)	**0.5** **(0.4, 0.7)**	**0.5** **(0.4, 0.7)**	70.8% (65.8, 75.8)	**0.6** **(0.4, 0.8)**	69.9% (62.5, 77.2)	**0.5** **(0.3, 0.7)**
Only attracted to opposite gender	82.0% (80.4, 83.6)	Reference	Reference	80.8% (78.2, 83.3)	Reference	83.0% (81.0, 85.1)	Reference
High competence							
Sexual minority	72.8% (68.6, 77.0)	**0.5** **(0.4, 0.7)**	**0.5** **(0.4, 0.7)**	71.5% (66.2, 76.8)	**0.5** **(0.4, 0.7)**	75.3% (68.4, 82.1)	**0.6** **(0.4, 0.9)**
Only attracted to opposite gender	83.5% (81.9, 85.1)	Reference	Reference	83.8% (81.3, 86.3)	Reference	83.3% (81.1, 85.4)	Reference
High relatedness							
Sexual minority	82.7% (79.4, 86.0)	**0.6** **(0.5, 0.8)**	**0.6** **(0.5, 0.8)**	84.5% (80.7, 88.2)	0.7 (0.5, 1.02)	79.4% (73.1, 85.6)	**0.5** **(0.3, 0.8)**
Only attracted to opposite gender	88.5% (87.1, 89.8)	Reference	Reference	88.4% (86.2, 90.6)	Reference	88.5% (86.8, 90.2)	Reference
	***M*** **(95% LCL, 95% UCL)**	**Unadjusted *b*** **(95% LCL, 95% UCL)**	**Adjusted* b*** **(95% LCL, 95% UCL)**	***M*** **(95% LCL, 95% UCL)**	**Unadjusted *b*** **(95% LCL, 95% UCL)**	***M*** **(95% LCL, 95% UCL)**	**Unadjusted *b*** **(95% LCL, 95% UCL)**
Mean life satisfaction							
Sexual minority	6.9 (6.8, 7.1)	** − 0.9** **(− 1.1, − 0.7)**	** − 0.8** **(− 1.0, − 0.6)**	6.8 (6.6, 7.0)	** − 0.9** **(− 1.1, − 0.6)**	7.2 (6.9, 7.6)	** − 0.8** **(− 1.1, − 0.4)**
Only attracted to opposite gender	7.9 (7.8, 7.9)	Reference	Reference	7.7 (7.5, 7.8)	Reference	8.0 (7.9, 8.1)	Reference

Results are weighted. The unadjusted models did not include any covariates. The adjusted models controlled for sex, visible minority status, household income quintile, and place of residence. Statistically significant odds ratios with 95% confidence intervals that do not include 1.0 are bolded. Statistically significant linear regression coefficients with 95% confidence intervals that do not include 0 are bolded. The sample sizes ranged from 5022 to 5044 for the overall results, 2567 to 2569 for the female results, and 2465 to 2473 for the male results due to varying rates of missing responses. *b*, unstandardized linear regression coefficient; *CHSCY*, Canadian Health Survey on Children and Youth; *LCL*, lower confidence limit; *M*, mean; *OR*, odds ratio; *UCL*, upper confidence limit

Results for specific sexual minority groups are presented in Table 3. Youth who were attracted to both males and females reported significantly lower average life satisfaction (6.9; *b* =  − 0.9; 95% CI, − 1.1 to − 0.7) and were significantly less likely to report high self-rated mental health (31.6%; OR = 0.3; 95% CI, 0.2 to 0.3), happiness (42.9%; OR = 0.5; 95% CI, 0.4 to 0.6), autonomy (70.9%; OR = 0.5; 95% CI, 0.4 to 0.7), competence (73.2%; OR = 0.5; 95% CI, 0.4 to 0.7), and relatedness (82.5%; OR = 0.6; 95% CI, 0.5 to 0.8) than youth who were only attracted to the opposite gender. In contrast, youth who were only attracted to the same gender were significantly less likely to report high autonomy (62.6%; OR = 0.4; 95% CI, 0.2 to 0.8) and competence (65.3%; OR = 0.4; 95% CI, 0.2 to 0.9) compared to youth with an exclusively heterosexual attraction, but did not significantly differ from them on any of the other PMH outcomes.

**Table 3:** Positive mental health outcomes in youth aged 15–17 years by specific sexual minority attraction versus exclusively heterosexual attraction, overall, 2019 CHSCY

Positive mental health outcome	Prevalence (95% LCL, 95% UCL)	Unadjusted OR (95% LCL, 95% UCL)
High self-rated mental health		
Only attracted to same gender	46.3% (29.3, 63.3)^C^	0.5 (0.2, 1.003)^C^
Attracted to both males and females	31.6% (27.2, 36.0)	**0.3** **(0.2, 0.3)**
Only attracted to opposite gender	63.6% (61.6, 65.6)	Reference
High happiness		
Only attracted to same gender	51.4% (34.3, 68.5)^C^	0.7 (0.3, 1.4)^C^
Attracted to both males and females	42.9% (38.3, 47.6)	**0.5** **(0.4, 0.6)**
Only attracted to opposite gender	61.5% (59.5, 63.6)	Reference
High autonomy		
Only attracted to same gender	62.6% (46.4, 78.7)	**0.4** **(0.2, 0.8)**
Attracted to both males and females	70.9% (66.7, 75.2)	**0.5** **(0.4, 0.7)**
Only attracted to opposite gender	82.0% (80.4, 83.6)	Reference
High competence		
Only attracted to same gender	65.3% (47.9, 82.7)	**0.4** **(0.2, 0.9)**
Attracted to both males and females	73.2% (68.9, 77.6)	**0.5** **(0.4, 0.7)**
Only attracted to opposite gender	83.5% (81.9, 85.1)	Reference
High relatedness		
Only attracted to same gender	86.6% (75.8, 97.4)	0.8 (0.3, 2.5)
Attracted to both males and females	82.5% (79.1, 85.9)	**0.6** **(0.5, 0.8)**
Only attracted to opposite gender	88.5% (87.1, 89.8)	Reference
	***M*** **(95% LCL, 95% UCL)**	**Unadjusted *b*** **(95% LCL, 95% UCL)**
Mean life satisfaction		
Only attracted to same gender	7.4 (6.8, 8.1)	− 0.4 (− 1.0, 0.2)
Attracted to both males and females	6.9 (6.7, 7.1)	** − 0.9** **(− 1.1, − 0.7)**
Only attracted to opposite gender	7.9 (7.8, 7.9)	Reference

Results are weighted. Statistically significant odds ratios with 95% confidence intervals that do not include 1.0 are bolded. Statistically significant linear regression coefficients with 95% confidence intervals that do not include 0 are bolded. The sample sizes ranged from 5034 to 5044 due to varying rates of missing responses. *b*, unstandardized linear regression coefficient; *CHSCY*, Canadian Health Survey on Children and Youth; *LCL*, lower confidence limit; *M*, mean; *OR*, odds ratio; *UCL*, upper confidence limit

^C^Interpret estimate with caution because coefficient of variation is greater than 15 and less than or equal to 25

### PMH and gender modality

Comparisons of each PMH outcome between gender minority youth versus cisgender youth are included in Table 4. Gender minority youth reported significantly lower average life satisfaction (6.0 versus 8.0; *b* =  − 2.0; 95% CI, − 2.9 to − 1.2) and were significantly less likely to report high self-rated mental health (34.0%^D^ versus 66.4%; OR = 0.3; 95% CI, 0.1 to 0.6^D^), happiness (32.7%^D^ versus 64.5%; OR = 0.3; 95% CI, 0.1 to 0.6^D^), autonomy (49.1%^C^ versus 81.0%; OR = 0.2; 95% CI, 0.1 to 0.5^C^), competence (54.1%^C^ versus 84.5%; OR = 0.2; 95% CI, 0.1 to 0.4^C^), and relatedness (70.1% versus 89.5%; OR = 0.3; 95% CI, 0.1 to 0.6) than cisgender youth.

**Table 4:** Positive mental health outcomes by gender modality in youth aged 12–17 years, overall, 2019 CHSCY

Positive mental health outcome	Prevalence (95% LCL, 95% UCL)	Unadjusted OR (95% LCL, 95% UCL)
High self-rated mental health		
Gender minority	34.0% (16.2, 51.7)^D^	**0.3** **(0.1, 0.6)**^D^
Cisgender	66.4% (65.3, 67.5)	Reference
High happiness		
Gender minority	32.7% (15.6, 49.8)^D^	**0.3** **(0.1, 0.6)**^D^
Cisgender	64.5% (63.3, 65.7)	Reference
High autonomy		
Gender minority	49.1% (32.3, 66.0)^C^	**0.2** **(0.1, 0.5)**^C^
Cisgender	81.0% (80.0, 81.9)	Reference
High competence		
Gender minority	54.1% (37.6, 70.6)^C^	**0.2** **(0.1, 0.4)**^C^
Cisgender	84.5% (83.5, 85.4)	Reference
High relatedness		
Gender minority	70.1% (55.3, 84.9)	**0.3** **(0.1, 0.6)**
Cisgender	89.5% (88.7, 90.2)	Reference
	***M*** **(95% LCL, 95% UCL)**	**Unadjusted *b*** **(95% LCL, 95% UCL)**
Mean life satisfaction		
Gender minority	6.0 (5.1, 6.9)	** − 2.0** **(− 2.9, − 1.2)**
Cisgender	8.0 (8.0, 8.1)	Reference

Results are weighted. Statistically significant odds ratios with 95% confidence intervals that do not include 1.0 are bolded. Statistically significant linear regression coefficients with 95% confidence intervals that do not include 0 are bolded. The sample sizes ranged from 11,023 to 11,046 due to varying rates of missing responses. *b*, unstandardized linear regression coefficient; *CHSCY*, Canadian Health Survey on Children and Youth; *LCL*, lower confidence limit; *M*, mean; *OR*, odds ratio; *UCL*, upper confidence limit

^C^Interpret estimate with caution because coefficient of variation is greater than 15 and less than or equal to 25

^D^Interpret estimate with caution because coefficient of variation is greater than 25 and less than or equal to 35

## Discussion

While much research attention has been devoted to examining negative psychological outcomes among SGM youth, less has been published documenting well-being among these populations. This study fills this gap with nationally representative data on numerous PMH outcomes among SGM youth in Canada in 2019. Overall, the results indicate that although PMH is not uncommon among SGM youth, significant disparities in PMH exist compared to exclusively heterosexual attracted youth and cisgender youth, respectively.

The size of some of the disparities was large. For instance, almost double the percentage of exclusively heterosexual attracted youth and cisgender youth reported high self-rated mental health compared to SGM youth. Moreover, the differences in mean life satisfaction were of a similar magnitude (for sexual minority compared to exclusively heterosexual attracted youth) or larger (for gender minority compared to cisgender youth) than the differences in mean life satisfaction observed in youth in Canada from the least versus most affluent households (Cosma et al., 2023). These findings reinforce adolescence as a potentially vulnerable period for poor mental health among some SGM individuals (Plöderl & Tremblay, 2015; Wittlin et al., 2023). SGM youth who are not reporting high levels of PMH may find themselves in home, school, and community contexts that are less supportive and where rejection, isolation, and prejudice are more commonplace (Blais et al., 2015; Ceatha et al., 2021; Wilson & Cariola, 2020). Indeed, SGM youth are more likely than cisgender heterosexual youth in Canada to report experiencing various types of bullying (Prokopenko & Hango, 2022). To minimize the observed disparities in PMH, it is likely necessary for interventions to target not just individual-level outcomes and determinants, but also broader risk and protective factors in the environments that SGM youth inhabit (e.g., a whole-of-school approach that promotes an inclusive school environment; McDermott et al., 2023).

When results were broken down by specific minority sexual attractions, we observed that youth who were attracted to both males and females significantly differed from youth who were only attracted to the opposite gender across all six PMH outcomes, while youth who were only attracted to the same gender significantly differed from the latter group on only two PMH outcomes. Based on data of those aged 15 years and older from the 2015 Canadian Community Health Survey, Gilmour (2019) also found that bisexual but not gay/lesbian individuals had a significantly lower prevalence of overall PMH compared to heterosexual individuals. The double discrimination and prejudice toward bisexuality (e.g., denying the validity of a bisexual orientation, questioning the reliability of bisexual partners) present in both heterosexual and gay/lesbian communities may partly explain why disparities were more consistently observed among youth who were attracted to both males and females (Friedman et al., 2014). Similarly, we found consistent disparities across all PMH outcomes for gender minority youth when compared to cisgender youth. Exposure to transphobic attitudes and behaviours, as well as experiences of gender dysphoria, expectations of stigma, and the stress of identity concealment/disclosure, may contribute to the lower odds of PMH among gender minority youth (Frost & Meyer, 2023; Tebbe & Budge, 2022).

### Strengths and limitations

The major strengths of this study include the documentation of disparities across a diversity of PMH outcomes among both SGM youth based on relatively recent data from a nationally representative population health survey. In addition, we were able to show that disparities frequently persisted between sexual minority youth and exclusively heterosexual attracted youth even after accounting for several sociodemographic factors. As the 2SLGBTQ + population is disproportionately young (Statistics Canada, 2021b), our focus on youth provides essential information on the PMH of an important segment of this population.

In terms of limitations, the relatively small number of SGM youth in the 2019 CHSCY dataset resulted in some estimates with high sampling variability and may have limited statistical power to detect smaller differences in PMH, particularly for youth who were exclusively attracted to the same gender. The limited sample size also restricted our ability to examine more specific SGM groups and stratify more results by additional sociodemographic factors. The interaction of multiple marginalized characteristics likely has implications for the well-being of SGM youth and warrants further research (Public Health Agency of Canada, 2022). The findings are only generalizable to youth within the target population of the 2019 CHSCY; the magnitude of disparities may have differed with the inclusion of youth from additional contexts (e.g., individuals living in institutions or homeless). While the reasons posited for the observed disparities are based on the existing literature, the analyses that we conducted in this study did not investigate the mechanisms by which these disparities emerge or protective/risk factors that may lead some SGM youth to report high versus lower levels of PMH (e.g., family support/rejection; Ceatha et al., 2021; Tebbe & Budge, 2022). The self-reported responses to survey questions may have been subject to bias and misclassification; for instance, some youth may have been unwilling to honestly disclose their sexual attraction or gender modality. Moreover, we excluded youth who indicated that they were not sure about their sexual attraction from our analyses; future studies could explore PMH among this group of youth who are questioning and still developing an understanding of their sexual orientation. To be consistent with language used in Wang et al. (2023), we also referred to gender when describing the different sexual attraction groups, but the survey question on sexual attraction did not distinguish between sex at birth and gender. The response options of “male” and “female” were included for both the sex at birth and gender identity survey questions, but are more appropriate for the former than for the latter (Rioux et al., 2022). Finally, the 2019 CHSCY only measured one of several aspects of sexual orientation (i.e., attraction, identity, and behaviour).

## Conclusion

This study provides evidence for numerous positive mental health outcomes differing by the sexual orientation and gender modality of youth in Canada. Disparities were most consistently observed among youth who were a gender minority or attracted to both males and females. While this highlights SGM youth as potential targets for mental health promotion and broader initiatives, the strength-based focus on PMH in this research also reveals how well-being was still being reported by many SGM youth. The results provide an important pre-pandemic baseline to investigate whether disparities in PMH may have widened or shrunk over time for SGM youth (e.g., with data from the second cycle of the CHSCY).

## Contributions to knowledge

What does this study add to existing knowledge?

- Previous research on the mental health of sexual and gender minority youth has tended to focus on negative psychological outcomes.
- This study documents disparities in positive mental health outcomes (i.e., average life satisfaction and high self-rated mental health, happiness, autonomy, competence, and relatedness) among gender and sexual minority youth in Canada in 2019 compared to their peers who are cisgender or exclusively attracted to the opposite gender, respectively.

What are the key implications for public health interventions, practice, or policy?

- The findings highlight sexual and gender minority youth as important populations for mental health promotion interventions.
- Broader initiatives that address risk and protective factors in the environments that sexual and gender minority youth inhabit are also likely critical in reducing these disparities.
- Continued public health surveillance of the positive mental health of sexual and gender minority youth is needed to examine whether these disparities have changed after 2019.

## Data Availability

The questionnaire is available on this Statistics Canada webpage: https://www23.statcan.gc.ca/imdb/p3Instr.pl?Function=assembleInstr&a=1&&lang=en&Item_Id=1209093. The data are available at Research Data Centres: https://www.statcan.gc.ca/en/microdata/data-centres.

## Code availability

The code is available from the authors on request.
